# Inactivation of viruses by coherent excitations with a low power visible femtosecond laser

**DOI:** 10.1186/1743-422X-4-50

**Published:** 2007-06-05

**Authors:** KT Tsen, Shaw-Wei D Tsen, Chih-Long Chang, Chien-Fu Hung, T-C Wu, Juliann G Kiang

**Affiliations:** 1 Department of Physics, Arizona State University, Tempe, AZ 85287, USA; 2 Department of Pathology, Johns Hopkins School of Medicine, Baltimore, MD 21231, USA; 3 Department of Oncology, Johns Hopkins School of Medicine, Baltimore, MD 21231, USA; 4 Departments of Obstetrics and Gynecology, Johns Hopkins School of Medicine, Baltimore, MD 21231, USA; 5 Departments of Molecular Microbiology and Immunology, Johns Hopkins School of Medicine, Baltimore, MD 21231, USA; 6 Scientific Research Department, Armed Forces Radiobiology Research Institute, Uniformed Services University of The Health Sciences, Bethesda, MD 20889-5603, USA; 7 Department of Medicine, Uniformed Services University of The Health Sciences, Bethesda, MD 20889-5603, USA; 8 Department of Pharmacology, Uniformed Services University of The Health Sciences, Bethesda, MD 20889-5603, USA

## Abstract

**Background:**

Resonant microwave absorption has been proposed in the literature to excite the vibrational states of microorganisms in an attempt to destroy them. But it is extremely difficult to transfer microwave excitation energy to the vibrational energy of microorganisms due to severe absorption of water in this spectral range. We demonstrate for the first time that, by using a visible femtosecond laser, it is effective to inactivate viruses such as bacteriophage M13 through impulsive stimulated Raman scattering.

**Results and discussion:**

By using a very low power (as low as 0.5 nj/pulse) visible femtosecond laser having a wavelength of 425 *nm *and a pulse width of 100 fs, we show that M13 phages were inactivated when the laser power density was greater than or equal to 50 *MW/cm*^2^. The inactivation of M13 phages was determined by plaque counts and had been found to depend on the pulse width as well as power density of the excitation laser.

**Conclusion:**

Our experimental findings lay down the foundation for an innovative new strategy of using a very low power visible femtosecond laser to selectively inactivate viruses and other microorganisms while leaving sensitive materials unharmed by manipulating and controlling with the femtosecond laser system.

## Background

Modern biochemical and pharmaceutical methods for inactivating or altering the functionality of viruses and other microorganisms are not only partially successful but also evoke problems of drug resistance and clinical side effects. New methods are therefore desirable, in particular for treating diseases such as HIV and other blood-borne viral diseases.

One experimental technique toward this goal is microwave absorption. However, a major drawback for excitation through microwave absorption is that water absorbs severely in the vibrational frequencies of capsids of viruses which are typically in the range of 30 GHz to 300 GHz [1,2]. Thus, it is extremely difficult to transfer microwave excitation energy to the vibrational energy of microorganisms. To overcome this difficulty, it is essential to transfer the excitation sources from the microwave range to, say, the visible range in which water is transparent.

Impulsive Stimulated Raman Scattering (ISRS) has been shown to be a viable way of producing large-amplitude vibrational modes in molecules in liquid solution as well as lattice vibrations in solid state systems [3]. In this paper, we report for the first time the use of a visible femtosecond laser system to excite a coherent acoustic Raman-active vibrational mode (which is associated with vibrations of viral capsids) in M13 phages through ISRS to such a high-energy state as to lead to their inactivation. Our work demonstrates a new method of manipulating, controlling, and inactivating unwanted microorganisms. It suggests that the basic principles of impulsive coherent excitation using a laser optical system can represent a general way to selectively alter the function of viruses and potentially other microorganisms through the property of their mechanical acoustic excitations. In addition, since structural change due to the mutation of microorganisms leads to very minimal variation of the vibrational frequency of their capsids, damage caused to viruses and/or other microorganisms through vibration of their mechanical structures likely would not be immune to simple mutation of cell surface receptors and the same treatment procedure remains valid, our approach would thus not evoke problems of drug resistance and as a result would be applicable to drug resistant strains of microorganisms.

## Sample and Experimental setup

The M13 bacteriophage samples used in this work were purchased from Stratagene.

**Figure 1:**
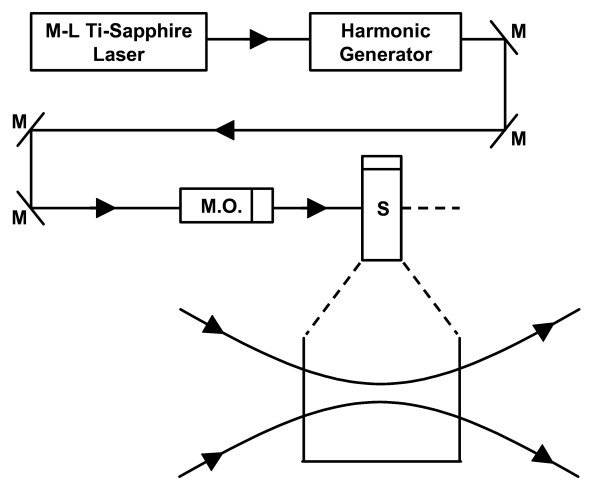
Experimental setup for the inactivation of M13 bacteriophages. M: mirror; M.O.: lens with extra long focus length; S: sample. The magnification shows the sample area where the laser beam is relatively uniform-focused. The cylindrical volume where the laser beam focuses defines the active volume for the inactivation of M13 bacteriophages through ISRS process.

The excitation source employed in this work is a diode-pumped cw mode-locked Ti-sapphire laser. The laser produces a continuous train of 80 fs pulses at a repetition rate of 80 MHz [4-7]. As shown in Figure 1, the output of the second harmonic generation system (SHG) of the Ti-sapphire laser is used to irradiate the sample. The excitation laser is chosen to operate at a wavelength of *λ *= 425 nm and with an average power of about 40 mW unless otherwise specified. It has a pulse width of full-width at half maximum (FWHM) ≅ 100 *fs*. A special lens of extra long focus length (*f *= 36 *cm*) is used to focus the laser beam into the sample area. The relatively uniformed laser-focused volume, which is the active volume for the interaction of laser with M13 bacteriophage through ISRS, very much approximates a cylinder having approximately 100 *μm *in diameter and 1.5 cm in height. In order to facilitate the interaction of laser with M13 bacteriophages which are inside a Pyrex cuvette and diluted in 2 ml water, a magnetic stirrer is set up so that M13 bacteriophages will enter the laser-focused volume as described above and interact with the photons. All the laser-irradiated M13 bacteriophage samples contained 1 × 10^7 ^*pfu/ml*. The assays were performed on the laser-irradiated samples after proper dilution. The typical exposure time of the sample to laser irradiation was about 10 hours. We believe that the amount of time (10 hours) required reflects the particular arrangement of our experimental setup and is not related to the efficiency of the inactivation of M13 bacteriophages by the laser system. In fact, our preliminary results indicated that a more efficient mixing arrangement resulted in a much shorter time required for the observation of inactivation of M13 bacteriophages. A thermal couple is used to monitor the temperature of the sample to ensure that our results are not due to the heating effects. We have found that the increase of the temperature of the M13 bacteriophage samples is less than 3°*C *after 10 hours' laser irradiation. All the experimental results reported here are obtained at T = 25°*C *and with the single laser beam excitation.

The activity of M13 bacteriophages was determined by plaque counts. In brief, M13 bacteriophages with nominally prepared 1 × 10^3 ^*pfu *or 5 × 10^2 ^*pfu *were added into a tube of soft agar at 70°C containing 0.3 ml of bacteria culture. Here, "nominally prepared" means that we prepare/dilute the M13 bacteriophage samples based on the *pfu *concentration specified by the manufacturer upon purchasing. The mixture was mixed well by vortexing and then poured onto a LB agar plate immediately. The plate was swirled well in order to spread the mixture over the entire plate evenly. The mixture on the agar plate was incubated for 8–16 hr. The plaques formed on the plate were counted.

All data are expressed as mean ± SD. Student's t-test was used for comparison of group with 5% as significant level.

## Experimental results, analysis and discussions

**Figure 2:**
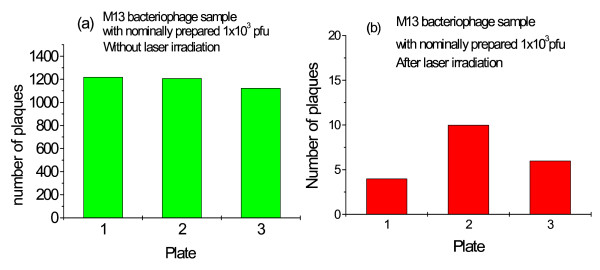
The three typical assays for a sample with nominally prepared 1 × 10^3 ^*pfu *of M13 bacteriophages (a) without laser irradiation; (b) after laser irradiation for about 10 hours. The extremely few number of plaques observed in the irradiated sample is a manifestation of almost complete inactivation of the M13 bacteriophages in the sample.

**Figure 3:**
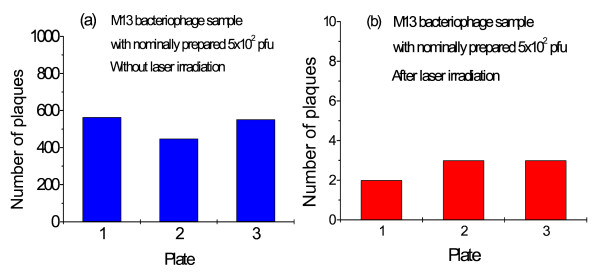
The three typical assays for a sample with nominally prepared 5 × 10^2 ^*pfu *of M13 bacteriophages (a) without laser irradiation; (b) after laser irradiation for about 10 hours. The extremely few number of plaques observed in the irradiated sample is indicative of almost complete inactivation of the M13 bacteriophages in the sample.

Fig. 2(a) shows the number of plaques of three typical assays for a sample with nominally prepared 1 × 10^3 ^*pfu *of M13 bacteriophages without the laser irradiation. The number of plaques is determined to be 1184 ± 52 counts. Fig. 2(b) shows the corresponding runs after the laser irradiation. It shows that the number of plaques after laser irradiation is 7 ± 3 counts (P < 0.0001 vs. sham-treatment; t = 39.14, df = 4) Similar results for another M13 bacteriophage sample with nominally prepared 5 × 10^2 ^*pfu *are shown in Fig. 3(a) and 3(b). The number of plaques in this case is determined to be 521 ± 64 counts for the sample without laser irradiation, and 3 ± 1 counts with laser irradiation (P < 0.0001 vs. sham-treatment; t = 14.02, df = 4). The intriguing feature is that there is very minimal amount of plaques for the laser irradiated samples as compared with the reference samples, indicative of the inactivation of M13 bacteriophages by the laser irradiation. We attribute the observed inactivation of M13 bacteriophages to laser-driven coherent excitations through ISRS process.

ISRS has been successfully demonstrated in molecular as well as solid state systems [8-12]. Yan et al. [3] predicted that ISRS should occur with no laser intensity threshold even when only one ultrashort laser pulse past through many types of media. In this case, ISRS is a forward-scattering process which is stimulated because the Stokes frequency is contained within the spectral width of the excitation pulse. Furthermore, they demonstrated that ISRS was a process through which excitation of coherent lattice or molecular vibrations would take place whenever a sufficiently short laser pulse past through a Raman-active solid or molecular liquid or gas. For a single beam excitation, if the damping is ignored, then the amplitude (*R*_0_) of the displacement away from the equilibrium intermolecular distance caused by ISRS can be shown to be given by [3]



where *I *is the power density of the excitation laser; *α *is the polarizability of the medium; *R *is the displacement away from the equilibrium intermolecular distance; *δα*/*δR *is proportional to the Raman scattering cross section; *ω*_0 _is the angular frequency of the excited coherent vibrational mode; *τ*_*L *_is the FWHM of the pulse width of the excitation laser; *m *is the molecular mass; *n *is the index of refraction; and c is the speed of light.

An intriguing aspect implied from Eq. (1) worth mentioning is that: For the one beam excitation experiment employed in this work, the primary beam as well as the Stokes beam, whose photon energies are denoted by *ħω*_*L *_and *ħω*_*s*_, respectively, define the excited coherent vibrations with energy *ħω*_0 _such that *ħω*_0 _= *ħω*_*L *_- *ħω*_*s*_. As a result, the FWHM of the spectral width of the excitation laser has to be larger than the energy of the excited coherent vibrations, which, because of the Gaussian distribution of the excitation laser in both time and space and by using uncertainty principle, gives rise to the factor:  in Eq. (1). This exponential dependence indicates that the product of angular frequency of the excited coherent vibration (*ω*_0_) and the FWHM of the excitation pulse width (*τ*_*L*_) has to be small in order that the amplitude *R*_0 _of the excited coherent vibration can be significant, i.e., *ω*_0_*τ*_*L *_≤ 1. This explains why the excitation laser should be ultrashort in pulse width for ISRS experiments. It also explains why the longer excitation laser pulse, even with the same power density, produces less inactivation as shown in our experimental results of Table 1 in which the dependence of the status of M13 bacteriophage on the excitation laser pulse width is presented.

**Table 1:** Dependence of the status of M13 bateriophages on pulse width (The laser power density is kept at 64 MW/cm**2)

Pulse width (fs) Status	100	250	500	600	700	800	1000	1500
Inactivation (Yes or No)	yes	yes	yes	yes	yes	no	no	no

From Eq. (1) it is clear that larger Raman cross sections, higher laser power densities, as well as lower vibrational frequencies, would contribute to bigger excited vibrational amplitude. In fact, for a moderate Raman scattering cross section, a sufficiently low vibrational frequency and a reasonable excitation power density, the amplitude of the vibrational displacement in the 0.01 to 1Å could be achieved through ISRS.

In our previous Raman scattering experiments [13,14], we have reported the observation of low frequency (≅ 8.5 *cm*^-1^) vibrational mode of M13 bacteriophages, which is shown to be associated with the axial torsion vibrations of the capsid of M13 bacteriophage. This particular Raman-active mode was observed because of relatively low damping rate resulting from the nature of its vibrations, i.e., it vibrates along the axis of the cylindrical-shape M13 bacteriophage. Therefore, we believe what is happening is that under our current ultrashort pulse laser excitation experiments with M13 bacteriophages, the amplitude of this mode has been coherently excited by ISRS to the extent that leads to their inactivation. To further support this explanation, we have carried out similar experiments with M13 bacteriophages by varying the power density as well as pulse width of the excitation laser.

**Figure 4:**
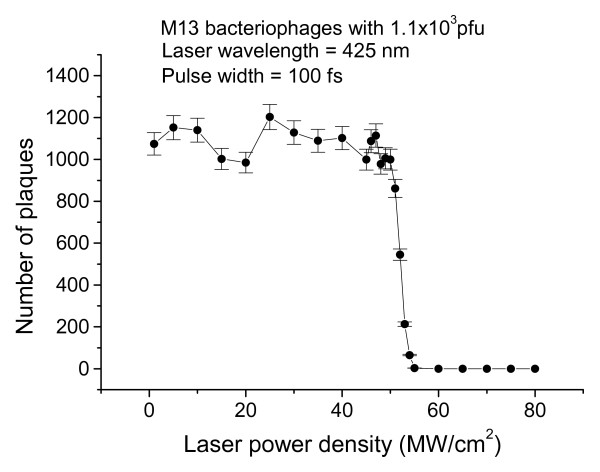
The number of plaques as a function of the excitation laser power density for a M13 bacteriophage sample with nominally prepared 1.1 × 10^3 ^*pfu*. The sharp cut-off for the number of plaques at around 50 *MW/cm*^2 ^is indicative of the onset of the inactivation of the M13 bacteriophages.

Fig. 4 shows the number of plaques as a function of the laser power density for M13 bacteriophage samples with 1.1 × 10^3 ^*pfu *after being irradiated with an excitation laser having 100fs- pulse width and *λ *= 425 nm. It is very interesting to observe an abrupt inactivation of the M13 bacteriophages at an excitation laser power density of about 50 *MW/cm*^2^. This observation is indicative of the fact that the M13 bacteriophages become inactivated as the amplitude of the vibrations exceeds a certain threshold. The reason why the M13 bacteriophages were inactivated at certain threshold amplitude is not clear at this moment. More work related to this area of research is needed.

We have also found that within the statistical error of the experiments there is almost no observable inactivation of the M13 bacteriophages if the pulse width of the excitation laser is longer than about 800fs while the power density of the excitation laser remains constant at ≅ 64 *MW/cm*^2 ^(this power density corresponds to an ultrafast laser with 100 fs pulse width and 40 mW of average power). The experimental results are summarized in Table 1. According to Eq. (1), if the laser power density remains constant, the amplitude of vibrational displacement excited by an ultrashort laser decreases with the increasing laser pulse width. As a matter of fact, Eq. (1) predicts that the amplitude of vibrational displacement excited by an ultrashort laser having a pulse width of 700 fs with power density of ≅ 64 *MW/cm*^2 ^approximately equals to that by an excitation laser having 100 fs pulse width but with power density 50 *MW/cm*^2^. Therefore, the experimental results in Table 1 are consistent with the predictions by Eq. (1) and with the power-density results of Fig. 4. We also notice that because the laser energy per pulse in the case of 800 fs or longer pulse width is actually higher than that in the case of 700 fs or shorter, the experimental results in Table 1 rule out the possibility that our observed inactivation of M13 bacteriophages is due to transient, micro-thermal effects that might develop at the laser focused volume.

We notice that viruses have been known to be inactivated by the ultraviolet photons with wavelengths around 250 nm. There is a possibility that our excitation laser might be generating 212.5 nm through the nonlinear property of the sample system; as a result the produced ultraviolet photons inactivate the M13 bacteriophages. However, this possibility can be ruled out. Because if the excitation laser with a pulse width of 100 fs and power density of 64 *MW/cm*^2 ^inactivated the M13 bcteriophages by the generated ultraviolet photons, then a laser with longer pulse width but the same power density would have produced more ultraviolet photons and would have been able to inactivate the M13 bacteriophages as well. This is in contradiction with the experimental results of Table 1. Thus, it is unlikely that the nonlinearly generated ultraviolet photons (if there are any) play a significant role in the inactivation of M13 bacteriophages observed in our current experiments.

Finally, we note that partly because our experimental results on power density and pulse width dependences are consistently explained by ISRS based upon the assumption that single laser pulse inactivates the M13 bacteriophage and partly because the fact the pulse separation produced in the laser system is as long as 13 ns, we believe that one single pulse in our visible femtosecond laser is capable of inactivating the M13 bacteriophage.

## Conclusion

We have shown that microorganisms such as bacteriophage M13 can be inactivated by a very lower power visible femtosecond laser system through ISRS. Our work demonstrates a new strategy for manipulating, controlling, and inactivating unwanted microorganisms. It suggests that the basic principles of impulsive coherent excitation using a laser optical system can be a general way to selectively alter the function of viruses and potentially other microorganisms through the property of their mechanical acoustic excitations. Furthermore, since damage caused to viral organisms through vibration of their mechanical structures likely would not trigger an immune response to simple mutation of cell surface receptors and the same treatment procedure remains valid, our approach would thus not evoke the problems of drug resistance. As a result, it would be applicable to drug resistant strains of microorganisms as well.
